# Tuberculosis in the European Region

**DOI:** 10.1007/s40475-023-00287-8

**Published:** 2023-05-27

**Authors:** Jessica Mencarini, Michele Spinicci, Lorenzo Zammarchi, Alessandro Bartoloni

**Affiliations:** 1 SOD Malattie Infettive e Tropicali, AOU Careggi, Florence, Italy; 2 Dipartimento di Medicina Sperimentale e Clinica, Unità Malattie Infettive, Università degli Studi di Firenze, Largo Brambilla 3, 50134 Florence, Italy

**Keywords:** Tuberculosis, COVID-19, Ukraine, Multi drug resistance, Pretomanid, Bedaquiline

## Abstract

**Purpose of Review:**

Tuberculosis (TB) remains a major public health concern worldwide. The COVID-19 pandemic and migration flow due to socioeconomic conditions, climate change, and geopolitical issues, such as the war, are important drivers influencing TB epidemiology in Europe. This article aims to review the data about TB in this area and the new findings about treatment and prevention strategies.

**Recent Findings:**

During the COVID-19 pandemic, access to health facilities and retention in care were difficult for TB patients, with consequences on TB diagnosis and TB incidence. The ongoing conflict in Europe, in areas with high prevalence of TB and MDR-TB, has reduced the access to health services and the availability of anti-TB drugs and increased the migration of refugees. New data on short treatment regimens could change the length of therapy and adverse events.

**Summary:**

TB control programs are facing emerging challenges that could change TB incidence in the near future. Novel antibiotic strategies and improved preventive policies could offer new opportunities to reduce the TB impact on public health.

## Introduction

Tuberculosis (TB) is a communicable disease and still remains a leading cause of death and disability, despite great efforts in prevention strategies and the recent development of new drugs. Globally, in 2021, there were an estimated 1.4 million deaths among HIV-negative people and an additional 187 000 deaths among people living with HIV. In 2019, WHO estimated that TB was the 13th leading cause of death worldwide and the top cause from a single infectious agent. In 2020, TB is the second leading cause of death from a single infectious agent, after COVID-19 [1–4].

Although TB is treatable and preventable, the roadmap towards TB control and elimination remains unclear. It is clear that TB elimination will require improved access to health care services and interventions to address well-recognized determinants of health that adversely affect TB outcomes, such as poverty, malnutrition, HIV infection, smoking, and diabetes.

Since 2014, the World Health Organization (WHO)’s End TB Strategy includes goals such as a reduction in the TB incidence rate, the absolute number of TB deaths, and the costs faced by TB patients and their households. In 2018, during the United Nations (UN) General Assembly, the goals were reaffirmed and the global targets for the funding for TB prevention, care, and research were set (see Box 1) [1, 2].

Since 2020, the achievement of TB targets was stopped by the COVID-19 pandemic that have caused a reduced access to care for TB. Along with COVID-19, other external factors, such as economic crisis, political instabilities, and human mass displacements, have negatively impacted on the health systems, with indirect effects on TB control strategies [4–6].

Box 1 [1, 2]

**Table:** 

**End the Global TB Epidemic** Percentage reduction in the absolute number of TB deaths: target for 2035 95% (compared with 2015 baseline) Percentage reduction in the TB incidence rate: target for 2035 90% (compared with 2015 baseline) Percentage of TB-affected households facing catastrophic costs due to TB **Indicator Target for End TB and for Global funding** Number of people with TB disease treated in the five years 2018–2022: 40 million people, including 3.5 million children and 1.5 million people with drug-resistant TB, including 115 000 children Number of people provided with TB preventive treatment in the five years 2018–2022: at least 30 million people Annual funding for universal access to quality prevention, diagnosis, treatment and care of TB: at least US$ 13 billion per year by 2022 Annual funding for TB research: US$ 2 billion annually in the five years 2018–2022	

## TB Epidemiology in Europe

In 2019, in 29 European Union (EU) and European Economic Area (EEA) countries, 49,752 TB cases have been reported with a notification rate of 9.6 per 100 000 population. Of them, 38,267 (76.9%) were newly diagnosed. In 2019, adults aged between 25 and 64 years accounted for 65.3% of all new and relapse TB cases, while children under 15 years accounted for 4.1% of all new and relapse TB cases. In 2020, an estimated 231 000 new and relapse TB cases (range 201 000–264 000) occurred in the WHO European Region, equivalent to an average incidence of 25 cases (range 22–28) per 100 000 population.

In addition, an estimated 21 000 TB deaths occurred among HIV-negative people in the European Region in 2020, equivalent to 2.3 deaths per 100 000 population (range 2.2–2.4). Between 2019 and 2020, data did not show a decline of the rate of TB mortality but a plateau, as a consequence of undiagnosed and untreated TB and an associated increase in deaths due to disruptions of TB services caused by the COVID-19 pandemic. Variation was seen across the region, ranging from fewer than one TB death per 100 000 population in western European countries up to 10 per 100 000 in some countries, such as Turkmenistan (10 deaths per 100 000), Tajikistan (9.6), and Ukraine (9.4).

TB patients of foreign origin represent 33% of all TB cases notified in EU/EEA countries and represent a large majority in several countries: Liechtenstein (100%), Malta (97.9%), Iceland (91.7%), Sweden (86.6%), Cyprus (86.1%), Luxembourg (85.3%), Israel (84.8%), Norway (81.3%), the Netherlands (72.4%), Denmark (72.1%), Switzerland (71.3%), and the UK (70.1%) [7, 8].

## Impact of COVID-19 and Other External Factors

At the global level, new diagnosis of TB dropped from 7.1 million in 2019 to 5.8 million in 2020. In 2021, WHO reported about 6.4 million of new TB diagnosis, a partial recovery. The WHO estimates that 4.1 million people are affected by TB, but they are undiagnosed or not reported to the national authorities. The regions of southeast Asia and the Western Pacific accounted for most of the global reduction in notifications of new TB cases. However, the decline in the TB incidence observed in the WHO European Region suggests that also this region was affected by the COVID-19 pandemic in terms of new TB diagnosis and notifications [1].

In the next years, this trend could lead to an increase of TB deaths, a reduction of multi-drug-resistant (MDR) TB treatment (−15%, from 177 100 to 150 350 MDR treatment), and a prevention treatment of latent tuberculosis (−21%, from 3.6 million to 2.8 million). The reduction of TB diagnosis and reporting is probably due to the difficulty to access to health facilities and retention on care for lockdowns and associated restrictions on movement, and similar symptoms of TB and COVID-19. Moreover, COVID-19 has caused a reallocation of resources and laboratory activities with a high impact on TB laboratory services and research activities, also in Europe [1, 6, 9].

More recently, the occurrence of a war in the heart of Europe is going to fuel TB concern in that area. Ukraine has one of the world’s highest burdens of MDR TB. According to the European Centre for Disease Prevention and Control (ECDC) data, in 2019, Ukraine reported 28 539 TB cases and an incidence of 65 cases per 100 000 with a mortality rate of 7.3 deaths per 100 000. In 2019, Ukraine reported 27% MDR-TB among new cases (4 490 cases) and 7 800 cases of HIV/TB coinfection (the second highest prevalence in the WHO European Region) [7, 8].

The current conflict has destroyed health facilities and roads, reducing the access to health services and the availability of anti-TB drugs.

Furthermore, the migration of refugees could be responsible of the spread of infectious diseases including TB in Europe, and an increase of MDR TB. Concern to infectious diseases outbreaks in the country and in the neighboring countries is very high and only the collaboration between authorities and medical organizations could prevent the lost of TB patients, ensuring therapy and early diagnosis of new cases of TB [10•, 11•, 12]. The ECDC recommended to European states to warrant health care services for refugees to early diagnosis of infectious disease as TB, especially MDR-TB, and to continue TB treatment [13, 14]. UNAIDS expects about 1 000–2 000 Ukrainian refugees with TB, requiring treatment and early diagnosis, and calls for special attention to avoid outbreaks or MDR emerging [11•].

## Immigration and TB in Europe

Immigration from high-incidence TB countries continues to be a driver for the potential increase of TB cases, cost of treatment, contact tracing, and medical issue to control TB and the emergence of MDR-TB. The main migrant groups include refugees, refused asylum seekers, and victims of human trafficking. So, the poor social conditions of migrants and the lack of access to health services increase the risk of TB and MDR-TB. Screening for latent TB and for active TB can reduce the incidence of TB and TB reactivation in this population and ensure early treatment [15, 16]. The potential correlation between migration and TB in Europe was recently analyzed by using available surveillance data: the study showed a statistically significant positive linear correlation between TB notification rates and immigration numbers for Germany, Italy, and Norway and a statistically significant negative correlation for Croatia, Estonia, Greece, Hungary, Ireland, Portugal, Slovakia, and the UK. Overall, the study did not establish a correlation between TB transmission and immigration, reducing concern to immigration risk [17].

## Treatment of Drug-Susceptible Tuberculosis

The early treatment of TB reduces the disease severity, the risk of transmission, and the rise of drug resistance. For drug-susceptible TB, the preferred regimen consists an intensive phase of 2 months of isoniazid, rifampin, pyrazinamide, and ethambutol followed by a continuation phase of 4 months of isoniazid and rifampin [18]. In 2021, the WHO Guideline Development Group reviewed data of the efficacy and safety of a 4-month regimen of rifapentine, isoniazid, pyrazinamide, and moxifloxacin for the treatment of drug-susceptible TB. The regimen is an alternative to the standard 6-month regimen, but the use is hampered by the availability and cost of rifapentine [19–21].

## Multidrug Resistant TB

The MDR-TB is defined as the resistance of *Mycobacterium tuberculosis* strains to at least isoniazid and rifampicin, the cornerstone drugs for the treatment of TB. Rifampicin-resistant (RR) disease requires similar clinical management as MDR-TB.

Recently, WHO announces updated definitions of extensively drug-resistant tuberculosis (XDR-TB). XDR-TB is caused by *M. tuberculosis* strains that fulfill the definition of MDR/RR-TB and are also resistant to any fluoroquinolone and at least one additional group A drug (group A drugs are the most potent group of drugs in the ranking of second-line medicines for the treatment of drug-resistant forms of TB and comprise levofloxacin, moxifloxacin, bedaquiline, and linezolid) [22].

Drug-resistant TB is confirmed to be a public health concern, although, globally, the incidence is stable (3–4% for newly TB cases and 18–21% for previously treated TB) [1]. In the countries of the former Soviet Union, the proportion of drug resistance in previously treated TB is about 50%.

In 2020, in Europe, the drug sensitivity test is available for 14 997 cases, of them 566 (3.8%) had multi-drug-resistant (MDR) TB. The countries with the highest proportion of MDR-TB cases were Estonia (19.4%) and Lithuania (16.7%). Pre-XDR-TB was reported for 27.6% of 417 RR/MDR-TB cases tested for second-line drug susceptibility; 37 (45.1%) met the XDR-TB definition. The HIV status is known for 12 327 TB cases; of them, 515 (4.2%) were reported as people living with HIV. In the ECDC report, the treatment outcome of 28 985 TB notified cases in 2019 is as follows: 71.8% were treated successfully, 7.6% died, and 0.8% experienced treatment failure. Of 927 RR/MDR-TB cases notified in 2018 with a treatment outcome reported in 2020, 52.4% were treated successfully, 14.5% died and 10.5% experienced treatment failure. Treatment success was reported only for 38.5% of XDR-TB cases notified in 2017, while 15.4% of cases were reported to have died and 3.8% experienced treatment failure [7, 8].

Although the number of reported TB cases declined, the reported treatment success is below the WHO targets in European countries.

## New Therapeutic Perspective

The introduction of new drugs such as bedaquiline, delamanid, and pretomanid has changed the treatment of MDR/XDR TB, improved the treatment success, and reduced the length of therapy [22, 23•, 24]. Recently, in TB WHO guidelines, a new shorter oral regimens were proposed for patients with confirmed MDR/RR TB. A 6-month regimen with bedaquiline, pretomanid, linezolid (600 mg), and moxifloxacin is recommended for MDR/RR TB patients with confirmed pulmonary or extrapulmonary TB (except central nervous system Tb, osteoarticular or miliary TB), and without previous exposure to bedaquiline, linezolid, pretomanid, or delamanid. Two clinical trials showed that the regimen with a linezolid dose of 600 mg was more effective than the conventional longer treatment, without severe adverse events. Moreover, WHO guidelines include an alternative short regimen (9 months) for patients without a history of treatment with second-line TB drugs for more than 1 month and in whom resistance to fluoroquinolones has been ruled out. This regimen is not indicated for disseminated TB and severe extrapulmonary TB. The combined therapy includes bedaquiline for 6 months, levofloxacin/moxifloxacin, ethionamide, ethambutol, isoniazid (high-dose), pyrazinamide, and clofazimine for 4 months (extended to 6 months if the sputum smear is positive at the end of 4 months), followed by 5 months of treatment with levofloxacin/moxifloxacin, clofazimine, ethambutol, and pyrazinamide. This regimen was associated with a high treatment success rate and a low rate of loss to follow up [25••]. The results of the STREAM stage 2 trial, an international, multi-center, parallel-group, open-label, randomized, controlled phase III trial examining a shorter all-oral bedaquiline-containing regimen, confirmed the efficacy and safety of this regimen for treatment of MDR TB (Table 1) [26–30].

**Table 1:** New drug regimens

Study	Phase	Regimen	WHO 2022 recommendation
TB-PRACTECAL	2-3	BPaLM BPaLC BPaL	BPaLM more treatment success, fewer failures/recurrence and less drug resistance than BPaL
ZeNix	3	BPa plus L (4 arms with different dosage and duration: L 1200 mg 26ws; 1200 9ws; 600 mg 26 ws; 600 mg 9 ws)	BPaL 600 mg 26 ws conditional for intervention
STREAM stage 2	2-3	Safety B plus multidrug therapy in 9 months and 6 months regimens	Superior to a 9-month injectable-containing regimen

*B*, bedaquilina; *Pa*, pretomanid; *L*, linezolid; *M*, moxifloxacina; *C*, clofazimine; *ws*, weeks

Pretomanid (Pa) is a nitroimidazole prodrug that undergoes nitroreduction within the mycobacterial cell. The primary mechanism of activity is inhibition of mycolic acid biosynthesis, like delamanid, but it has lower protein binding and higher tissue penetration than delamanid. The metabolites of pretomanid induce also the killing of latent bacteria. Pa was approved in 2019 by the FDA and in 2020 by the European Medicines Agency (EMA), both as part of a 6-month regimen containing bedaquiline (B) and linezolid (L; BPaL regimen) for treatment of XDR TB, and treatment intolerant or non-responsive MDR TB. In the studies, the regimen showed a favorable outcome 6 months after treatment completion and sustained favorable outcome after 24 months; also, due to the adverse events correlated to linezolid, the trial that compares different doses of linezolid, had showed improving of tolerability with reduced linezolid dosing with high cure rate [31–33]. Currently, about 16 compounds for TB treatment are in the early clinical development phase, thanks to genetic approaches that could detect new potential drug target. New oxazolidinone as sutezolid (PNU-100480), delpazolid (LCB01-0371), and TBI-223 showed promising antimycobacterial activity; also, delpazolid (LCB01-0371) and TBI-223 have lower potency against mitochondrial protein synthesis, and a shorter half-life than linezolid, which may improve mitochondrial toxicity [34–37]. New targets and new compounds have been identified, and new clinical trials will change the treatment of TB (Table 2).

**Table 2:** Targets of new compounds

Mechanism of action	New compounds
23S rRNA binding	Sutezolid, Delpazolid, TBI-223
Inhibitor of aminoacyl-tRNA synthetases	GSK656
Cell wall synthesis inhibition	TBA-7371, BTZ-043, PBTZ-169, OPC-167832, SQ109
Electron transport chain inhibition	TBAJ-876, TBAJ-587, TBI-166, Telacebec (Phase 2a)
DNA synthesis inhibition	SPR720
Cholesterol catabolism inhibition	GSK2556286
Transcriptional regulators inhibition	BVL-GSK098

## Conclusions

TB remains a leading cause of death and disability worldwide. In Europe, COVID-19 and geopolitical issues negatively affected TB epidemiology, reducing TB diagnosis and reporting, as well as the access to health facilities, and increasing the risk of the emergence of MDR-TB. In the next year, the European health care services should guarantee an early diagnosis of infectious disease as TB, especially MDR-TB, and a retention in care for TB patients. The introduction of new drugs offers the possibility for shortening the length of the therapy and reducing the occurrence of adverse event, increasing the chance of treatment success.
